# Intravitreal Injection of Hydrogen Peroxide Induces Acute Retinal Degeneration, Apoptosis, and Oxidative Stress in Mice

**DOI:** 10.1155/2018/5489476

**Published:** 2018-11-08

**Authors:** Bing Huang, Jia-Jian Liang, Xi Zhuang, Shao-Wan Chen, Tsz Kin Ng, Haoyu Chen

**Affiliations:** ^1^Joint Shantou International Eye Center of Shantou University and the Chinese University of Hong Kong, Shantou, Guangdong, China; ^2^Shantou University Medical College, Shantou, Guangdong, China; ^3^Department of Ophthalmology and Visual Sciences, The Chinese University of Hong Kong, Hong Kong

## Abstract

**Purpose:**

Oxidative stress is a common pathological condition for multiple retinal diseases. Hydrogen peroxide (H_2_O_2_) has been applied as an oxidative stress inducer for the *in vitro* studies. Here, we report the *in vivo* effect of H_2_O_2_ exposure to the mouse retina and its underlying mechanism.

**Methods:**

The H_2_O_2_ or saline solution was intravitreally injected into the eyes of female C57BL/6J mice for two consecutive days. The retinal structure was evaluated by *in vivo* imaging using spectral domain optical coherence tomography (OCT) and validated by histological assessment as well as retinal marker expression. In addition, retinal stress, cell apoptosis, and antioxidant enzyme expression were also determined.

**Results:**

Retinal and outer nuclear layer thickness thinning was observed at days 7 and 14 by OCT imaging with the treatment of 10 *μ*g H_2_O_2_, which was confirmed by the histopathological analysis. The expressions of photoreceptor (*Rho*, *Rora*, *Rorb*, and *Rcvrn*), bipolar cell (*Chat* and *Calb2*), and retinal pigment epithelial (*Rpe65*) markers were reduced in the H_2_O_2_-treated group, whereas the expression of retinal ganglion cell marker (*Tubb3*) was increased. TUNEL-positive cells were obviously found in the outer nuclear layer and inner nuclear layer of H_2_O_2_-treated mice but sparely found in the ganglion cell layer. Coherently, apoptotic gene expressions (*Casp3*, *Casp9*, *Bax*, and *Parp8*) were significantly increased in the retina with increasing dosages of H_2_O_2_, while *Bcl2* expression was mildly decreased. In addition, the expressions of Gfap and antioxidant enzyme genes (*Txn2*, *Sod2*, and *Gpx4*) were significantly upregulated in the retina after the H_2_O_2_ treatment, compared to the vehicle control group.

**Conclusions:**

This study revealed that intravitreal injection of H_2_O_2_ induces acute retinal damage by increasing oxidative stress and cell apoptosis in the retina. This acute retinal degeneration mouse model could provide a platform for drug screening against oxidative stress and retinal diseases.

## 1. Introduction

Retinal diseases, including age-related macular degeneration (AMD), glaucoma, diabetic retinopathy (DR), and retinitis pigmentosa (RP), are the leading cause of irreversible blindness and visual impairment in most developed countries [1, 2], affecting more than 300 million people worldwide. Although intraocular pressure lowering can slow down the progression of glaucoma and photodynamic therapy and antivascular endothelial growth factor treatments are effective in neovascular AMD and DR, the retinal diseases still cannot be cured. Elucidating the disease mechanisms can facilitate the development of new treatments against the retinal diseases [3].

The common pathology in the retinal diseases is the retinal degeneration mediated by cell apoptosis [4, 5]. Antiapoptotic treatments have been proven to prevent the retinal cells from degeneration [6, 7]. Reactive oxygen species (ROS) induces oxidative stress through lipid peroxidation, disruption of normal mitochondrial function, and DNA damage, all of which can initiate the caspase-mediated apoptosis pathway [8]. Hydrogen peroxide (H_2_O_2_), as one of the ROS, has widely been used to induce cellular oxidative stress in different cell lines, including retinal pigment epithelial (RPE) and 611W cells [9, 10]. The *in vitro* cell culture can mimic the oxidative disease mechanisms for initial high-throughput drug screening; yet, the *in vivo* model would be more suitable for the development of antioxidative treatments before clinical trials [11, 12]. Intracameral injection of H_2_O_2_ has been shown to cause edematous ciliary process edema and deterioration as well as corneal endothelial damage [13]. However, the *in vivo* effect and mechanism of H_2_O_2_ on mammalian retina in experimental models have yet to be determined.

In the current study, we aimed to investigate the effect and mechanism of intravitreal injection of H_2_O_2_ in mice so as to establish an *in vivo* platform for drug screening against oxidative stress-related retinal diseases. The retinal structure and cell integrity were evaluated by *in vivo* imaging, histological assessment, and gene expression analysis. In addition, cell apoptosis and oxidative stress status in the H_2_O_2_-treated retina were also determined.

## 2. Material and Methods

### 2.1. Animals

Female C57BL/6J mice (7-week-old, about 20 grams) were purchased from Beijing Vital River Laboratory Animal Technology Co. Ltd., China. The mice were housed under standard conditions of 12 : 12 hour dark-light cycle with access to standard rodent chow and water *ad libitum*. For each experimental group, 3–4 mice were used for the experiments. All mice were treated according to the guidelines of the Association for Research in Vision and Ophthalmology (ARVO) Statement on Use of Animals in Ophthalmic and Vision Research. The study protocol was approved by the Animal Experimentation Ethics Committee of the Joint Shantou International Eye Center of Shantou University and the Chinese University of Hong Kong.

### 2.2. Intravitreal Injection of Hydrogen Peroxide

The H_2_O_2_ solution (catalog number: 88597; Sigma-Aldrich, St. Louis, MO) was diluted with saline to the final concentration of 5, 8, and 10 *μ*g/*μ*l. The mice were anesthetized with 1 : 1 mixture (1.5 ml/kg) of ketamine (100 mg/ml) and xylazine (20 mg/ml), and intravitreal injection with a posterior approach behind the corneoscleral limbus of the eyeball was conducted under a stereomicroscope (MZ 9.5; Leica, Germany) without damaging the lens. A prepulled glass pipette connected to a Hamilton syringe (Reno, NV) and prefilled with mineral oil (Sigma-Aldrich) was used for the H_2_O_2_ injection. On the stating day (day 0) and the day after (day 1), 1 *μ*l per eyeball of H_2_O_2_ solution or equal volume of saline control was intravitreally injected with a period over 2 minutes. At postinjection days 2, 7, and 14, the mice were sacrificed for further histological and gene expression analyses.

### 2.3. *In Vivo* Imaging

The combination of confocal scanning laser ophthalmoscope (cSLO) with spectral domain optical coherence tomography (OCT; RETImap animal, Roland, Germany) was used for *in vivo* imaging of the retina before and after the H_2_O_2_ injection. A superluminescent diode with a wavelength of 830 ± 50 nm was used as the laser source, and a maximum scan speed of 25,000 A-scan/sec was applied. A micrometer-resolution, three-dimensional imaging with infrared cSLO provides a planar visualization of the retina. The digital image depth of the cSLO was 16 frames per second with software module eye-tracking activated. The fundus photographs and OCT images were simultaneously captured on the exact retinal locus in a 30° circle surrounding the optic nerve head. These images were averaged automatically by the built-in software to augment the signal-to-noise ratio. The thicknesses of the retina, defined as the distance between the inner limiting membrane and Bruch's membrane, and the outer nuclear layer (ONL) were manually measured from each averaged OCT image at 600 *μ*m away from the edge of the optic disc. Two images were measured for each experimental mouse.

### 2.4. Histological Assessment of the Retina

The H_2_O_2_-treated mice were anesthetized and sacrificed by the perfusion with 0.9% saline followed by 4.0% paraformaldehyde in 0.1 M Na_2_HPO_4_/NaH_2_PO_4_ buffer (pH 7.4). The eyeballs were enucleated, post-fixed in 4.0% paraformaldehyde for 4 hours, and cryoprotected with 30% sucrose/PBS for 2 days. The eyeball slices (10 *μ*m) were sectioned using the vibratome (Leica), stained with hematoxylin and eosin, and imaged with the pupil-optic nerve position using a light microscope (Nikon, Japan). At 600 *μ*m from the edge of the optic nerve cup, the thicknesses of the retina, ONL, inner nuclear layer (INL), and inner plexiform layer (IPL) were measured, and the cell densities of each retinal layer were also counted [14]. Six sections were measured for each experimental mouse.

### 2.5. Retinal Stress and Apoptosis Analyses

Retinal stress was evaluated by the expression of glial fibrillary acidic protein (Gfap) by the immunofluorescence analysis with rabbit anti-Gfap antibody (1 : 400; Abcam, the United Kingdom) on the vibratome-sectioned eyeball slices (10 *μ*m). Apoptosis was evaluated using the TUNEL method coupled with fluorescein (DeadEnd™ Fluorometric TUNEL System kit, Promega, Madison, WI) and DAPI counterstain (1 : 2000; Sigma-Aldrich). Apoptotic cells were visualized under the Leica TCS SP5-II fluorescence confocal microscope and counted at 600 *μ*m from the edge of the optic nerve cup. Six sections were counted for each experimental mouse.

### 2.6. Gene Expression Analysis

The retina was dissected from the eyeball in the RNAlater (Invitrogen, Carlsbad, CA) immediately after enucleation. Total RNA was extracted from the retina using the TRIzol® reagent (Invitrogen) and reverse transcribed into complementary DNA with random primers using the Superscript First-Strand Synthesis System (TaKaRa, Japan). Gene expression analysis was performed using the Power SYBR Green PCR Master Mix (TaKaRa) with specific primers (Supplementary Table 1). The *Gapdh* gene was used as housekeeping gene for normalization.

### 2.7. Statistical Analysis

The data was represented as mean ± standard error of mean (SEM) and analyzed using Stata 14. The data distribution was analyzed by the Kolmogorov-Smirnov test. The means were compared using the independent *t*-test or one-way or repeated two-way analysis of variance (ANOVA) with Bonferroni post hoc test. Statistical significance was defined as *p* < 0.05.

## 3. Results

### 3.1. Hydrogen Peroxide Induced Acute Retinal Damages

**Figure 1:**
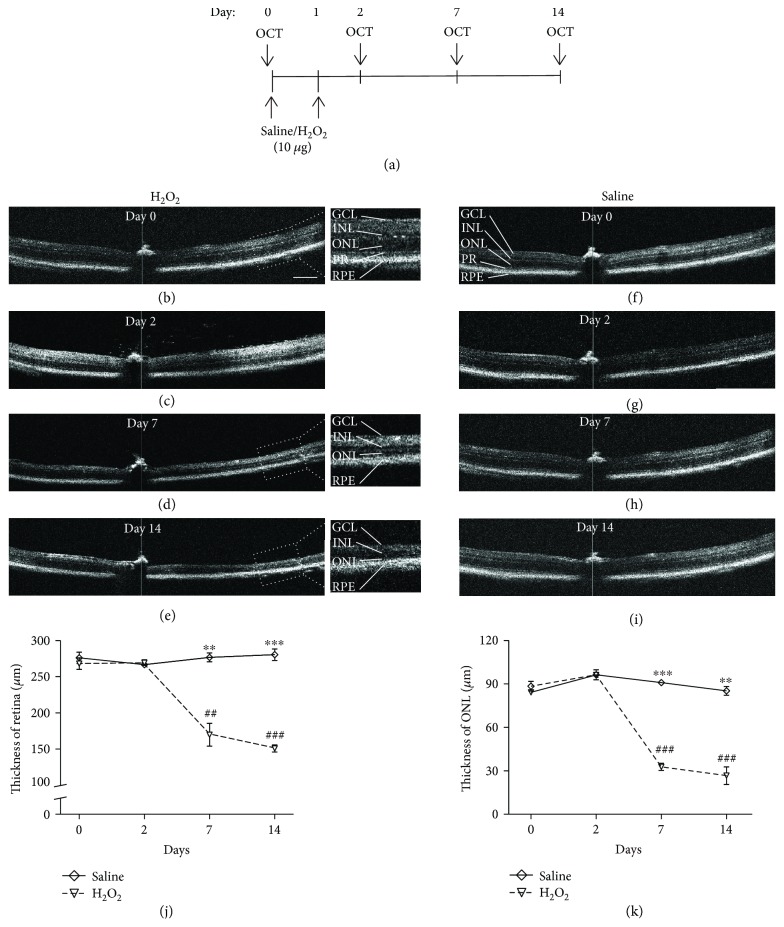
OCT analysis of the retina after the H_2_O_2_ treatment. (a) The schematic diagram of OCT analysis along the H_2_O_2_ treatment. (b–e) OCT images of the retina at days 0, 2, 7, and 14 of the H_2_O_2_ group. (f–i) OCT images of the retina at days 0, 2, 7, and 14 of the saline group. Retinal lesion was showed by a decreased in thickness of ONL and disruption of the PR both at days 7 and 14 after H_2_O_2_ exposure (d, e). (j) The total thickness of the retina showed a significant decrease after 7 days of H_2_O_2_ treatment and became more severe after 14 days. (k) The local thickness of the ONL also showed a significant decrease after 7 days of H_2_O_2_ treatment and more severe after 14 days (*n* = 6 from 3 mice). ^∗∗^ *p* < 0.01, ^∗∗∗^ *p* < 0.001 compared to H_2_O_2_; ^##^ *p* < 0.01, ^###^ *p* < 0.001 compared to day 0 within the H_2_O_2_ group. Scale bar: 200 *μ*m.

*In vivo* OCT images were taken at day 0 (baseline) before the intravitreal injection of H_2_O_2_ for two consecutive days and at postinjection days 2, 7, and 14 (Figure 1(a)). The OCT images at baseline and day 2 of the H_2_O_2_-treated mice showed the typical laminar structure of the retina (Figures 1(b) and 1(c)) without obvious changes in the retina (day 0: 267.99 ± 7.88 *μ*m and day 2: 269.32 ± 3.19 *μ*m; Figure 1(j)) and ONL thicknesses (day 0: 84.24 ± 1.71 *μ*m and day 2: 96.29 ± 0.76 *μ*m; Figure 1(k)), which were similar to the morphologies (Figures 1(f) and 1(g)) and thicknesses in the saline-treated group (retina: 275.998 ± 7.85 *μ*m at day 0 and 266.66 ± 0.83 *μ*m at day 2, Figure 1(j); ONL: 88.45 ± 3.29 *μ*m at day 0 and 96.30 ± 2.80 *μ*m at day 2, Figure 1(k)). Yet, the thickness of the photoreceptor inner segment ellipsoid zone (PR) was reduced at day 7 (Figure 1(d)) and more severely at day 14 (Figure 1(e)). Moreover, significant decrease in the thickness of the retina (day 7: 169.69 ± 15.76 *μ*m, *p* = 0.003 and day 14: 150.74 ± 4.72 *μ*m, *p* < 0.001; Figure 1(j)) and ONL (day 7: 32.78 ± 2.47 *μ*m, *p* < 0.001 and day 14: 26.72 ± 6.06 *μ*m, *p* < 0.001; Figure 1(k)) was also identified in the H_2_O_2_-treated mice, compared to that in the saline-treated control (retina: 276.58 ± 6.20 *μ*m at day 7 and 280.41 ± 7.93 *μ*m at day 14; ONL: 90.98 ± 0.71 *μ*m at day 7 and 85.28 ± 3.07 *μ*m at day 14, Figures 1(h) and 1(i)).

**Figure 2:**
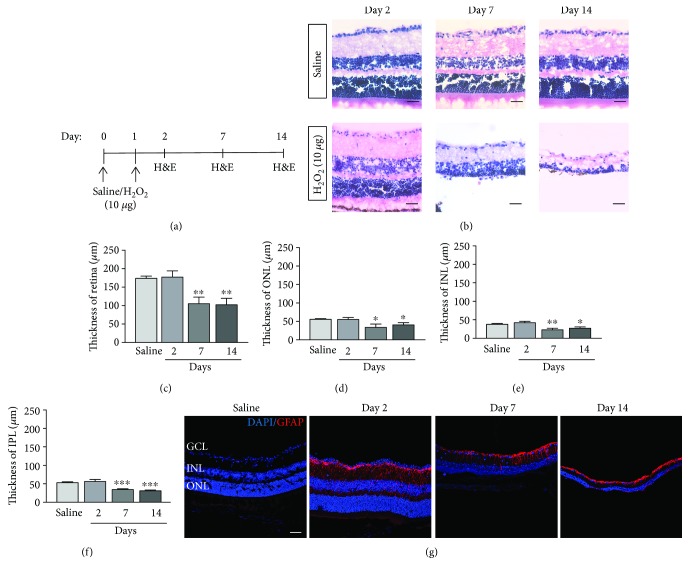
Histological assessment of the retina after the H_2_O_2_ treatment. (a) The schematic diagram of histological analysis after the H_2_O_2_ treatment. (b) Representative of the retinae stained by hematoxylin and eosin. (c–f) Thickness of the retina, ONL, INL, and IPL after the H_2_O_2_ treatment. (c) Thickness of the retina was decreased at postinjection days 7 and 14 but not day 2. (d) Thickness of the ONL was decreased at postinjection days 7 and 14 but not day 2. (e) Thickness of the INL was decreased at postinjection days 7 and 14 but not day 2. (f) Thickness of the IPL was decreased at postinjection days 7 and 14 but not day 2. (*n* = 5–6 from 3 mice). ^∗^ *p* < 0.05, ^∗∗^ *p* < 0.01, and ^∗∗∗^ *p* < 0.001 compared to the saline group. Scale bar: 200 *μ*m. (g) Glial fibrillary acidic protein (Gfap; red) expression in the retina after the H_2_O_2_ treatment.

To verify the results of *in vivo* imaging, the structure of the H_2_O_2_-treated retina was resolved by the hematoxylin and eosin staining (Figures 2(a) and 2(b)). At day 2, no alteration of retinal thickness was observed (retina: 177.20 ± 16.93 *μ*m, *p* = 0.841; ONL: 55.40 ± 5.33 *μ*m, *p* = 0.956; INL: 41.98 ± 3.84 *μ*m, *p* = 0.344; and IPL: 56.39 ± 12.24 *μ*m, *p* = 0.559, Figures 2(c)–2(f)). The retinal degeneration observed in the OCT imaging was confirmed by the histological assessment that, at days 7 and 14, the thicknesses of the retina (day 7: 105.01 ± 17.85 *μ*m, *p* = 0.004; day 14: 102.25 ± 17.45 *μ*m, *p* = 0.003, Figure 2(c)), ONL (day 7: 33.76 ± 9.12 *μ*m, *p* = 0.030; day 14: 40.74 ± 6.10 *μ*m, *p* = 0.032, Figure 2(d)), INL (day 7: 22.90 ± 4.06 *μ*m, *p* = 0.009; day 14: 27.16 ± 3.62 *μ*m, *p* = 0.032, Figure 2(e)), and IPL (day 7: 34.30 ± 5.27 *μ*m, *p* < 0.001; day 14: 32.17 ± 5.34 *μ*m, *p* < 0.001, Figure 2(f)) were significantly reduced after the H_2_O_2_ treatment when compared to the saline control (retina: 173.71 ± 6.19 *μ*m, ONL: 55.70 ± 1.89 *μ*m, and INL: 37.68 ± 2.31 *μ*m). The OCT and histology results indicated that intravitreal injection of H_2_O_2_ induces the thinning of the laminar structure in the retina. Furthermore, immunofluorescence analysis demonstrated a persistent increase in Gfap expression from days 2 to 14 after the H_2_O_2_ treatment (Figure 2(g)), indicating the induction of retinal stress by the H_2_O_2_ treatment.

**Figure 3:**
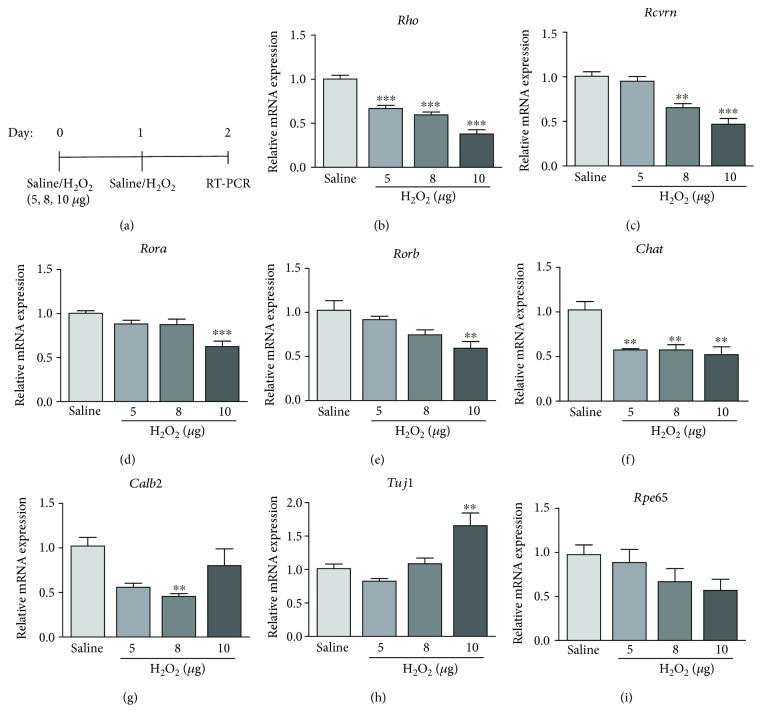
Retinal gene expression analysis after the H_2_O_2_ treatment. (a) The schematic diagram of gene expression analysis after the H_2_O_2_ treatment. The expression of photoreceptor markers including (b) *Rho*, (c) *Rcvrn*, (d) *Rora*, and (e) *Rorb* genes was decreased significantly with the increasing dose of H_2_O_2_. (f) The expression of *Chat* gene was decreased significantly after treatment with H_2_O_2_. (g) The expression of *Calb2* gene was decreased significantly after treatment with 8 *μ*g H_2_O_2_. (h) The expression of *Tuj1* gene was significantly increased in 10 *μ*g H_2_O_2_ group. (i) The expression of *RPE65* gene was similar to that in the control group. ^∗∗^ *p* < 0.01, ^∗∗∗^ *p* < 0.001, compared to the saline group (*n* = 5–6 from 3 mice).

To confirm the retinal cell damage induced by H_2_O_2_, gene expressions of retinal markers were determined after H_2_O_2_ application. For the photoreceptor cells, rhodopsin gene (*Rho*) expression decreased significantly with increasing doses of H_2_O_2_ (5 *μ*g: 0.67 ± 0.03 folds, 8 *μ*g: 0.60 ± 0.03 folds, and 10 *μ*g: 0.38 ± 0.05 folds, *p* < 0.001; Figure 3(b)), compared to the saline control. Similarly, the expressions of other photoreceptor genes [15], recoverin (*Rcvrn*; 8 *μ*g: 0.65 ± 0.05 folds, *p* < 0.01 and 10 *μ*g: 0.47 ± 0.06 folds, *p* < 0.001, Figure 3(c)), nuclear receptor ROR-alpha (*Rora*; 10 *μ*g: 0.62 ± 0.06 folds, *p* < 0.001, Figure 3(d)), and nuclear receptor ROR-beta (*Rorb*; 10 *μ*g: 0.59 ± 0.08 folds, *p* < 0.01, Figure 3(e)) genes, were also dose-dependently downregulated in the H_2_O_2_-treated retina, compared to the saline-treated retina. For the amacrine cells, the expression of choline acetyltransferase (*Chat*) was significantly reduced in all doses of H_2_O_2_ applications (5 *μ*g: 0.58 ± 0.01 folds, 8 *μ*g: 0.58 ± 0.06 folds, and 10 *μ*g: 0.52 ± 0.09 folds, *p* < 0.01; Figure 3(f)), compared to the saline control. For the retinal ganglion cells (RGCs) [16], the expression of calretinin gene (*Calb2*) was significantly lower in the 8 *μ*g H_2_O_2_-treated group than that of the saline control group by 0.46 folds (*p* < 0.01; Figure 3(g)); yet, the expression of the *β*III-tubulin gene (*Tubb3*) was significantly higher in the 10 *μ*g H_2_O_2_ group than that of the saline control group by 2.45 folds (*p* < 0.01; Figure 3(h)). Nonetheless, the expression of retinal pigment epithelium 65 gene (*Rpe65*) did not show significant changes among the treatment groups (Figure 3(i)). The gene expression results confirmed that the retinal degeneration begins immediately after the H_2_O_2_ application in mice.

### 3.2. Hydrogen Peroxide Induced Retinal Cell Apoptosis in a Dose-Dependent Manner

**Figure 4:**
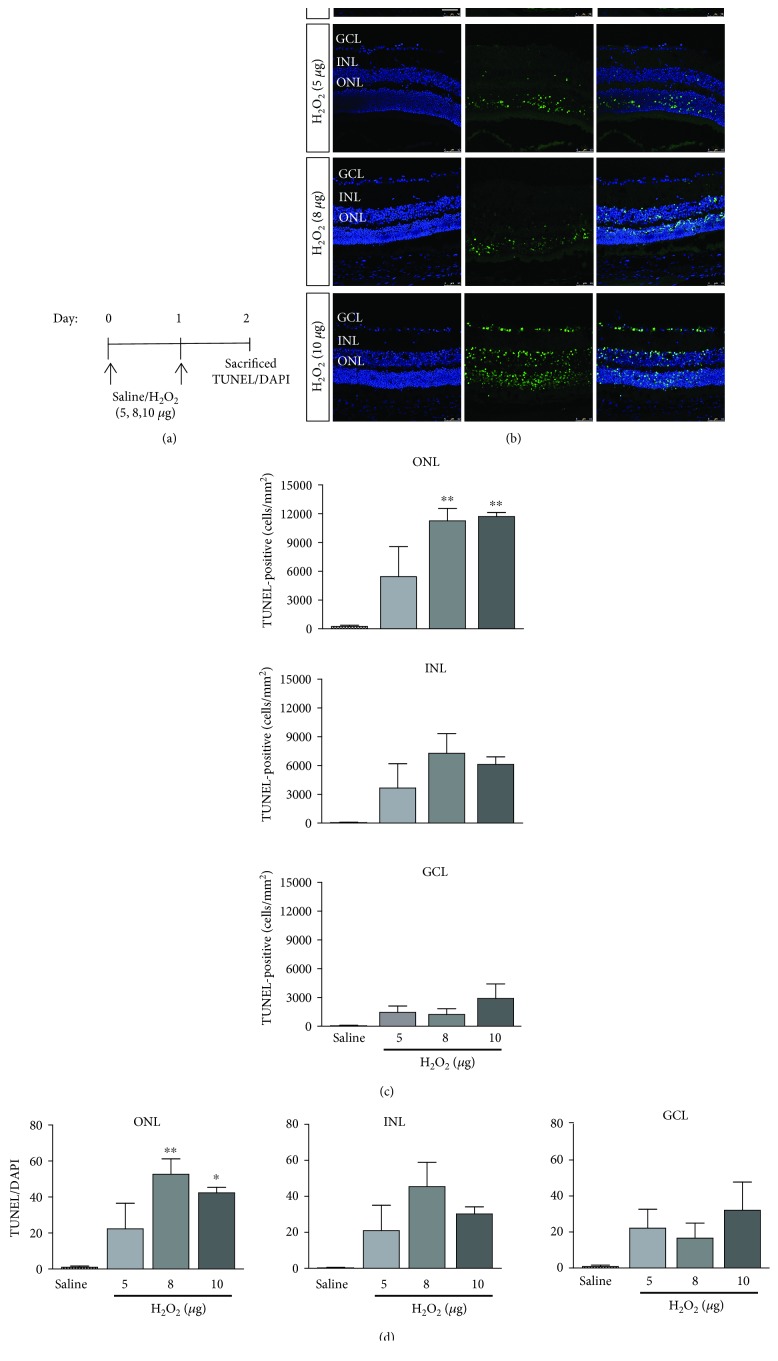
Apoptosis analysis in the retina after the H_2_O_2_ treatment. (a) The schematic diagram of apoptosis analysis after the H_2_O_2_ treatment. (b) No TUNEL-positive cells were found in the saline group. After H_2_O_2_ treatment, TUNEL-positive cells were found in both the ONL and INL in each group and sparely found in GCL in the 10 *μ*g H_2_O_2_ group. (c) The number of TUNEL-positive cells increased after the H_2_O_2_ treatment in the ONL, INL, and GCL. (d) The proportions of TUNEL-positive cells showed an increase after H_2_O_2_ treatment in the ONL, INL, and GCL. ^∗^ *p* < 0.05, ^∗∗^ *p* < 0.01 compared to the saline-treated group (*n* = 6 from 3 mice). Scale bar: 50 *μ*m.

To delineate the mechanisms of the H_2_O_2_-induced retinal cell damage, cell apoptosis in the retina was evaluated by the TUNEL assay. No TUNEL-positive cell was found in all retinal layers of the saline-injected mice (Figure 4(b)). In contrast, the TUNEL-positive cells were increased dose-dependently in all retinal layers. In the 10 *μ*g H_2_O_2_-induced group, 11689.15 ± 498.87 cells/mm^2^ of TUNEL-positive cells were found in the ONL (*p* = 0.003), 6108.11 ± 858.73 cells/mm^2^ in the INL (*p* = 0.121), and 2889.52 ± 834.59 cells/mm^2^ (*p* = 0.487) in the ganglion cell layer (GCL; Figure 4(c)). Comparatively, less TUNEL-positive cells were observed in the 5 and 8 *μ*g H_2_O_2_ injection (5 *μ*g: 3085.22 ± 1314.75 cells/mm^2^ in the ONL, *p* = 0.297; 1029.08 ± 289.01 cells/mm^2^ in the INL, *p* = 0.904; and 183.37 ± 99.51 cells/mm^2^ in the GCL, *p* = 1.000; 8 *μ*g: 6773.00 ± 698.94 cells/mm^2^ in the ONL, *p* = 0.006; 3743.66 ± 919.53 cells/mm^2^ in the INL, *p* = 0.072; and 437.08 ± 259.99 cells/mm^2^ in the GCL, *p* = 1.000).

**Figure 5:**
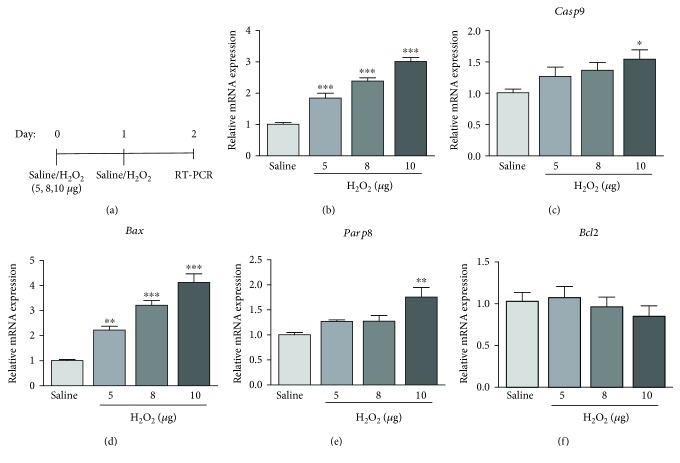
Expression analysis of apoptotic genes after the H_2_O_2_ treatment. (a) The schematic diagram of gene expression analysis after the H_2_O_2_ treatment. The expression of (b) *Casp3*, (c) *Casp9*, (d) *Bax*, and (e) *Parp8* genes was increased with increasing doses of H_2_O_2_. (f) The expression of *Bcl2* gene did not show a significant change after the H_2_O_2_ treatment. ^∗^ *p* < 0.05, ^∗∗^ *p* < 0.01, and ^∗∗∗^ *p* < 0.001, compared to the saline-treated group.

To verify the results of TUNEL assay on the H_2_O_2_-treated retina, the expression of apoptosis-related genes was examined. Coherent to the TUNEL results, the expressions of caspase-3 (*Casp3*; 5 *μ*g: 1.84 ± 0.14 folds, 8 *μ*g: 2.39 ± 0.10 folds, and 10 *μ*g: 3.01 ± 0.13 folds, *p* < 0.001; Figure 5(b)) and Bcl-2-associated X (*Bax*; 5 *μ*g: 2.29 ± 0.13 folds, *p* < 0.01, 8 *μ*g: 3.21 ± 0.19 folds, and 10 *μ*g: 4.12 ± 0.35 folds, *p* < 0.001; Figure 5(d)) genes were significantly increased in the H_2_O_2_-treated retina in a dose-dependent manner, compared to the saline-treated control (Figure 5(a)). In addition, the expressions of caspase-9 (*Casp9*; 1.54 ± 0.15 folds, *p* < 0.05; Figure 5(c)) and poly(ADP-ribose) polymerase family member 8 (*Parp8*; 1.75 ± 0.019 folds, *p* < 0.01; Figure 5(e)) genes were also significantly increased in the 10 *μ*g H_2_O_2_ group. Yet, the expression of the antiapoptosis gene *Bcl2* did not show statistically significant changes after the H_2_O_2_ application (Figure 5(f)). Collectively, the TUNEL assay and gene expression results indicated that the H_2_O_2_-induced retinal degeneration could be caused by cell apoptosis.

### 3.3. Hydrogen Peroxide Induced Oxidative Stress in the Retina

**Figure 6:**
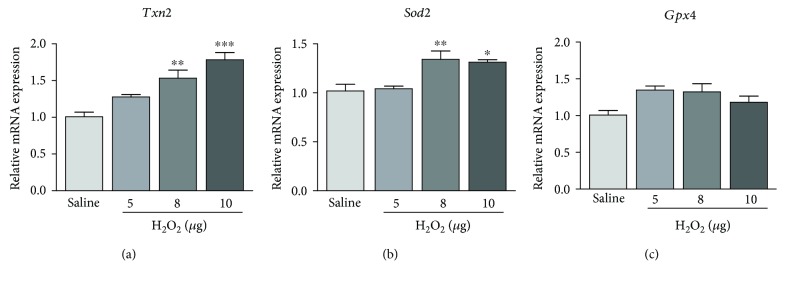
Expression analysis of oxidative stress-related genes after the H_2_O_2_ treatment. After H_2_O_2_ treatment, the expression of antioxidant genes (b) *Txn2*, (b) *Sod2*, and (c) *Gpx4* increased significantly between groups in the retina. ^∗^ *p* < 0.05, ^∗∗^ *p* < 0.01, and ^∗∗∗^ *p* < 0.001, compared to the saline-treated group.

In addition to cell apoptosis, the expression of antioxidant genes was determined. Compared to the saline control, the expression of thioredoxin-2 (*Txn2*) gene was significantly upregulated with increasing doses of H_2_O_2_ (8 *μ*g: 1.53 ± 0.11 folds, *p* < 0.01 and 10 *μ*g: 1.78 ± 0.10 folds, *p* < 0.001; Figure 6(a)). In addition, the application of 8 *μ*g and 10 *μ*g H_2_O_2_ also showed a significant elevation in superoxide dismutase 2 (*Sod2*) gene expression by 1.34 ± 0.09 folds (*p* < 0.01) and 10 *μ*g: 1.32 ± 0.02 folds (*p* < 0.05), respectively, compared to the saline control (Figure 6(b)). In contrast, the expression of glutathione peroxidase 4 (*Gpx4*) gene did not show statistically significant changes after the H_2_O_2_ application (Figure 6(c)). These suggested that the increased expression of antioxidant genes could be responded to the H_2_O_2_-induced oxidative stress elevation in the retina.

## 4. Discussion

In the current study, our results showed that (1) H_2_O_2_ exposure induces the thinning of the whole retina, ONL, and photoreceptor inner segment ellipsoid zone; (2) H_2_O_2_ exposure induces retinal cell apoptosis and reduces retinal cell density; and (3) H_2_O_2_ increases the oxidative stress in the retina.

Retinal cell death is a key pathology of retinal degenerative diseases, including AMD, glaucoma, RP, and DR. The underlying molecular mechanisms still remain elusive. The transgenic models, such as *rd1* or *rd10*, are useful to investigate the pathology and the disease mechanisms of retinal degeneration as they exhibit progressive retinal cell loss spontaneously [17]; yet, most of them have an early onset of degeneration, and the severity and the onset of the retinal degeneration cannot be modulated [3]. Instead, chemical-induced models can also be applied for the study of disease mechanisms and for the screening of potential treatments. N-Methyl-D-aspartate (NMDA) has been applied to induce RGC apoptosis through the NMDA receptor [18]. In this study, we injected H_2_O_2_ intravitreally to induce retinal degeneration in mice, and we found that H_2_O_2_ induces degeneration in all retinal layers (Figures 1 and 2), which the photoreceptor cells are the most severely damaged, followed by the cells in the INL. Interestingly, the expression of *β*III-tubulin gene (*Tubb3*) showed an increase in the 10 *μ*g H_2_O_2_ treatment (Figure 3), in which cell apoptosis and oxidative stress are significantly found. This could be explained by previous reports that overexpression of *β*III-tubulin represents a major mechanism of drug resistance to microtubule-interacting agents, such as taxanes and Vinca alkaloids, and *β*III-tubulin is conditionally expressed in A2780 cells after hypoxia [19]. Nevertheless, we demonstrated a dose-dependent retinal damage for the H_2_O_2_-induced model (Figures 3 and 4), indicating that the severity of retinal degeneration can be modulated and controlled. Besides, the reduction in IPL thickness and the increased Gfap expression (Figure 2) could resemble retinal stress and ischemia [20], which could be induced by the H_2_O_2_-mediated oxidative stress.

Previous studies reported that H_2_O_2_ induces apoptosis in RGCs *in vitro* and inhibits the phosphorylation of p38 and extracellular signal-regulated kinases1/2 [21]. The rabbit study also showed that intracameral injection of H_2_O_2_ causes morphological changes in the ciliary processes with upregulation of 3-aminotriazole, an inhibitor of catalase [13]. H_2_O_2_ has been found to be toxic to both the lens and cornea in high concentrations [22]. However, the *in vivo* effect of H_2_O_2_ exposure to the retina is still poorly understood. This study, for the first time, reported the effect of intravitreal injection of H_2_O_2_ in mouse retina. Based on the *in vivo* imaging and histological assessment, we confirmed that H_2_O_2_ induces acute retinal degeneration in mice, in which cell apoptosis begins right after the H_2_O_2_ application although the retina structure is still maintained (Figures 4 and 5).

Mitochondrial ROS triggers the release of cytochrome c and other proapoptotic proteins, which can trigger caspase activation and apoptosis [23]. H_2_O_2_ has been found to induce apoptosis in rat nucleus pulposus cells as well as PC12 cells through the mitochondria-mediated pathway [24, 25]. Coherently, we found that H_2_O_2_ exposure enhances the expression of the mitochondrial antioxidant genes (*Txn2* and *Sod2*) in the retina (Figure 6). Simultaneously, the mitochondrial apoptosis-related genes (*Casp3*, *Bax*, and *Parp8*) are upregulated in the H_2_O_2_-treated retina (Figure 5). Herein, we postulate that H_2_O_2_ induces mitochondrial oxidative stress, which in turn triggers the mitochondrial apoptotic pathway and leads to the retinal cell death and the structural disruption. Further investigations could focus on the mitochondrial oxidative stress-targeted pharmacological agents against the H_2_O_2_-induced retinal degeneration.

In summary, this study revealed that intravitreal injection of H_2_O_2_ induces acute retinal degeneration in mice by increasing oxidative stress and cell apoptosis. This acute retinal degeneration mouse model could provide a drug screening platform for oxidative stress and retinal diseases.

## Data Availability

The data used to support the findings of this study are available from the corresponding author upon request.

## Supplementary Materials

Supplementary Materials Supplementary Table 1: “the primers used in real-time PCR.”
