# Supplementary material for: Intravitreal Injection of Hydrogen Peroxide Induces Acute Retinal Degeneration, Apoptosis, and Oxidative Stress in Mice
Source: Oxid Med Cell Longev. 2018 Nov 8;2018:5489476. doi: 10.1155/2018/5489476 (PMC6250010; doi:10.1155/2018/5489476)
Supplement: Supplementary Materials — Supplementary Table 1: “the primers used in real-time PCR.” [file 5489476.f1.docx]

**Supplementary table 1**

| **Gene** |  | **Sequence (5’-3’)** | **Annealing Tm (°C)** | **Reference** |
| --- | --- | --- | --- | --- |
| *Bax* | F: | AGACAGGGGCCTTTTTGCTAC | 60 | NM_007527 |
|  | R: | AATTCGCCGGAGACACTCG |  |  |
| *Bcl2* | F: | GTGGATGACTGAGTACCT | 60 | NM_007546 |
|  | R: | CCAGGAGAAATCAAACAGAG |  |  |
| *Calb2* | F: | TCCTGCCGACCGAAGAGAAT | 60 | NM_009810 |
|  | R: | GCTTAGGTTCATCATAGGGCCTG |  |  |
| *Casp3* | F: | TGGTGATGAAGGGGTCATTTATG | 60 | NM_007527 |
|  | R: | TTCGGCTTTCCAGTCAGACTC |  |  |
| *Casp9* | F: | TCCTGGTACATCGAGACCTTG | 60 | NM_015733 |
|  | R: | AAGTCCCTTTCGCAGAAACAG |  |  |
| *Chat* | F: | GGCCATTGTGAAGCGGTTTG | 60 | NM_009891 |
|  | R: | GCCAGGCGGTTGTTTAGATACA |  |  |
| *Gapdh* | F: | GTGGAGTCATACTGGAACATGTAG | 60 | NM_008085 |
|  | R: | AATGGTGAAGGTCGGTGTG |  |  |
| *Gpx4* | F: | CCATGCACGAATTCTCAGCC | 60 | NM_008162 |
|  | R: | GTGACGATGCACACGAAACC |  |  |
| *Parp8* | F: | ACTGCCTGTTTGCAGATTTCA | 60 | NM_001081009 |
|  | R: | TGGACGGAGTAGGATACACTTTT |  |  |
| *Rho* | F: | CCCTTCTCCAACGTCACAGG | 60 | NM_001025086 |
|  | R: | TGAGGAAGTTGATGGGGAAGC |  |  |
| *Rora* | F: | GTGGAGACAAATCGTCAGGAAT | 60 | NM_013646 |
|  | R: | TGGTCCGATCAATCAAACAGTTC |  |  |
| *Rorb* | F: | GTTCGCCAAGCGGATAACAG | 60 | NM_146095 |
|  | R: | GGGTTGAAGGCACGACACAT |  |  |
| *Rpe65* | F: | ACCACTAACAGCTCATGTCACA | 60 | NM_029987 |
|  | R: | ACAGGTGATAGAAAGGCTCAGAT |  |  |
| *Rcvrn* | F: | ACGACGTAGACGGCAATGG | 60 | NM_009038 |
|  | R: | CCGCTTTTCTGGGGTGTTTT |  |  |
| *Sod2* | F: | CAGACCTGCCTTACGACTATGG | 60 | NM_013671 |
|  | R: | CTCGGTGGCGTTGAGATTGTT |  |  |
| *Tuj1* | F: | TAGACCCCAGCGGCAACTAT | 60 | NM_023279 |
|  | R: | GTTCCAGGTTCCAAGTCCACC |  |  |
| *Txn2* | F: | TGGGCTTCCCTCACCTCTAAG | 60 | NM_019913 |
|  | R: | CCTGGACGTTAAAGGTCGTCA |  |  |
